# Intracellular ATP Assay of Live Cells Using PTD-Conjugated Luciferase

**DOI:** 10.3390/s121115628

**Published:** 2012-11-12

**Authors:** Mi-Sook Lee, Wan-Soon Park, Young Han Kim, Won Gyeong Ahn, Seung-Hae Kwon, Song Her

**Affiliations:** 1 Division of Bio-Imaging, Chuncheon Center, Korea Basic Science Institute, Chuncheon 200-701, Korea; E-Mails: mslee0621@gmail.com (M.-S.L.); wans8@kbsi.re.kr (W.-S.P.); yhkim10@kbsi.re.kr (Y.H.K.); wgahn09@kbsi.re.kr (W.G.A.); kwonsh@kbsi.re.kr (S.-H.K.); 2 Acupuncture & Meridian Science Research Center, College of Oriental Medicine, Kyung Hee University, Seoul 130-701, Korea

**Keywords:** intracellular ATP, PTD-Conjugated luciferase, live cell-based assay, 2-deoxy- glucose, iodoacetic acid, potassium cyanide

## Abstract

Luciferase is a sensitive, reliable biological sensor used for measuring ATP. However, its widespread application in drug discovery and toxicology studies has been limited due to unavoidable cell extraction processes, which cause inaccurate measurements of intracellular ATP and obstruct the application of homogenous high-throughput screening. Recently, we developed a protein transduction domain-conjugated luciferase (PTD-Luc) for measuring cellular uptake efficacy. In this study, we evaluated the applicability of PTD-Luc to an intracellular ATP assay of live cells. The predominant fluorescence of Alexa 647-PTD-Luc was in the cytosol, whereas the fluorescence of Alexa 647-Luc was visualized surrounding the cell membrane, as confirmed by Western blot analysis. *In vitro*, PTD-Luc could detect less than 10^–9^ M ATP, and the correlation between the luciferase activity of PTD-Luc and the ATP content was strong (R = 0.999, *p* < 0.001). *In vivo*, luminescence signals of PTD-Luc detected intracellular ATP in as few as 50 HeLa cells, with a strong correlation between luminescence and cell number, suggesting high sensitivity and reliability. Furthermore, two blockers of the glycolytic pathway (2-deoxyglucose and iodoacetic acid) inhibited the signal in a dose-dependent manner, whereas potassium cyanide, an inhibitor of oxidative phosphorylation, had no effect on intracellular ATP *in vivo*, as seen with the PTD-Luc sensor. These data show that PTD-Luc can directly measure the intracellular ATP content in live cells, allowing real-time kinetic studies, suggesting that it is a promising tool for high-throughput drug screening and cytotoxicity assays.

## 1. Introduction

Assessing metabolic function via the cellular ATP content is a widely used method of measuring cellular toxicity in assays that are essential for screening drugs and testing toxicological safety. ATP monitoring can be also used to assess the cytocidal, cytostatic, and proliferative effects of a range of drugs, biological response modifiers, and biological compounds [1,2]. As ATP plays a central role in cellular metabolism, its intracellular level is regulated precisely in healthy cells. Moreover, early cell injury results in not only decreased ATP synthesis but also rapid depletion of endogenous ATP caused by the release of ATP-converting enzymes (e.g., ATPase) [3]. Therefore, rapid and accurate measurement of the intracellular ATP content is critical for determining the status of cellular toxicity.

Many methods have been used to measure ATP, but the most sensitive and reliable technique is a bioluminescent method based on the luciferin-luciferase reaction [4,5]. In this reaction, ATP powers the luciferase-mediated conversion of luciferin into oxyluciferin, which produces a chemiluminescent signal that is proportional to the amount of ATP, according to the following equation:

$$\text{ATP}+D-\text{Luciferin}+O_{2}^{-}+{\text{Mg}}^{2+}\overset{\text{Luciferase}}{\to }\text{Oxylucifein}+\text{AMP}+{\text{PP}}_{i}+{\text{CO}}_{2}+h\upsilon$$

The current bioluminescent ATP assays are necessary to release the intracellular ATP by the cell extraction processes including cell harvesting, lysis, and separation steps. However, the use of extraction substances, neutralization of extractants, and other procedures performed on extracted components adversely affect the cellular ATP content. Reduced membrane integrity caused by the extraction process also results in the rapid loss of cytoplasmic ATP, leading to inaccurate measurement of the intracellular ATP content and limiting the application of the method to homogenous high-throughput screening (HTS). Recently, to reduce the problems caused by cell extraction processes, methods have been developed that use modified cell lysis solutions, such as the single-step homogenous method [2,6]. Using an integrating reagent, this method shows high sensitivity, excellent linearity, simplicity, and rapidity, and requires no cell harvesting or separation steps. However, the direct detection of intracellular ATP and the need for additional processes are still unresolved.

We recently developed a protein transduction domain-conjugated luciferase (PTD-Luc) for measuring cellular uptake efficacy [7]. We demonstrated that PTD can penetrate the cell membrane without affecting its integrity or interfering with cellular metabolism. The luminescence of PTD-conjugated firefly luciferase should prove advantageous for many *in vivo* applications involving pharmaceuticals. Here, we evaluated the applicability of PTD-Luc as an ATP sensor for measuring the intracellular ATP content. The greatest advantage of PTD-Luc is that it allows the rapid, direct measurement of the intracellular ATP content in live cells without requiring cell extraction processes.

## 2. Experimental Section

### 2.1. Cell Culture

HeLa cells were obtained from the American Type Culture Collection (ATCC; Manassas, VA, USA) and grown in Dulbecco's modified Eagle's medium (DMEM) supplemented with 10% fetal bovine serum (FBS), 100 U/mL penicillin, and 100 U/mL streptomycin under 5% CO_2_ at 37 °C, as recommended by the supplier. To examine the ATP response to anti-metabolites, HeLa cells were co-cultured with the metabolic inhibitors 2-deoxyglucose (2-DG; Sigma, St. Louis, MO, USA), iodoacetic acid (IAA; Sigma), or potassium cyanide (KCN; Sigma) in glucose-free Ringer buffer (116 mM NaCl, 5.6 mM KC1, 0.8 mM MgSO_4_, 1 mM NaH_2_PO_4_, 1 mM KH_2_PO_4_, 4.8 mM NaHCO_3_, 1.8 mM CaCl_2_, and 20 mM HEPES, pH 7.2) [8].

### 2.2. ATP Assay

The firefly luciferase-conjugated protein transduction domain (YARVRRRGPRR, PTD-Luc) has been described, and luciferase (Luc) protein expression and purification were performed as described previously [7,9]. For the cell-free luciferase assay, luciferin substrate solution was prepared containing 1 mM d-luciferin (Biosynth International, Naperville, IL, USA) and 15 mM MgSO_4_ in 30 mM HEPES (pH 7.8) using fresh, deionized ATP-free water. ATP (ATP disodium salt; Sigma) was diluted serially in the luciferin substrate solution. Each 10 μL aliquot of PTD-Luc or Luc was combined with 90 μL of luciferin substrate solution. Immediately, the luciferase activity was acquired as an image using an IVIS-200 imaging system (xenogen Corp., Alameda, CA, USA).

To assess the intracellular ATP assay, HeLa cells were cultured to 70% confluence in black-walled 96-well microtiter plates. One hour before treatment with Luc or PTD-Luc, the culture medium was replaced with glucose-free Ringer buffer, and the cells were treated with 2-DG, IAA, or KCN at the concentrations indicated in the figures. Then, 40 nM Luc or PTD-Luc was incubated in the cell medium for 5 min, and d-luciferin was added to a final concentration of 150 μg/mL. Light emissions were acquired for approximately 30 s using an IVIS-200 system (xenogen Corp.) and quantified as photon flux (photons/s, p/s; using Xenogen Living Image^®^ software.

### 2.3. Fluorescence Confocal Microscopy

The purified PTD-Luc protein was labeled using an Alexa 647 protein labeling kit (Life Technologies, Grand Island, NY, USA), according to the manufacturer's protocol. Briefly, protein was incubated with Alexa 647 dye for 2 h at room temperature. After incubation, the free dye was removed using BioGel P-30 (40000 MWCO; Bio-Rad, Hercules, CA, USA). The protein labeling efficiency was determined using sodium dodecyl sulfate-polyacrylamide gel electrophoresis (SDS-PAGE) and analyzed using a Xenogen IVIS-200 imaging system (xenogen) to ensure the quality before use, as described previously [10]. For intracellular visualization, HeLa cells were incubated for 30 min in glucose-free Ringer solution containing 170 nM PTD-Luc-Alexa 647 at 37 °C in the presence of 5% CO_2_. After washing twice with PBS, the cells were fixed with 4% paraformaldehyde for 10 min and stained with Hoechst 33342 (Molecular Probes, Eugene, OR, USA) for 10 min at room temperature. Fluorescence signals were analyzed using a confocal laser scanning microscope (LSM-5 and LSM System, ver. 3.98; Carl Zeiss, Oberkochen, Germany).

### 2.4. Western Blot Analysis

Western blot analysis was carried out as described previously [7]. Protein concentrations were determined using a bicinchoninic acid (BCA) protein assay (Pierce Biotechnology, Rockford, IL, USA). The primary antibodies used were specific for luciferase (1:1,000; Sigma) or β-actin (1:10,000; Sigma). Immunoreactive proteins were detected using horseradish peroxidase-conjugated IgG (1:5,000; Santa Cruz Biotechnology, Santa Cruz, CA, USA) and visualized using an ECL kit (Amersham, Little Chalfont, Buckinghamshire, UK).

### 2.5. Statistical Analysis

Dose-response curve fitting and data analysis were performed using nonlinear regression with Prism 4 (GraphPad Software, San Diego, CA, USA). To detect statistical correlations, Pearson's correlation coefficient (R) was calculated between the luciferase activity and ATP concentration or cell number. Significant differences were detected using paired *t*-tests. Each result is represented as the mean ± SEM, * *p* < 0.05, ** *p* < 0.01.

## 3. Results and Discussion

**Figure 1.**
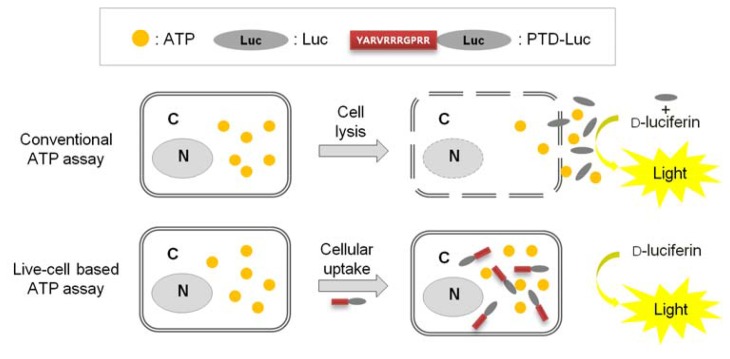
Schematic of the direct measurement of intracellular ATP using PTD-Luc in a live cell. Inset: PTD-Luc with 11 amino acids (YARVRRRGPRR) and Luc as a control. The conventional ATP assay requires cell lysis to release the cell contents, including ATP. By contrast, in the live-cell ATP assay, PTD-Luc can cross the cell membrane without perturbing the cell, enabling the direct measurement of the intracellular ATP content. C, cytosol; N, nucleus.

Previously, we reported that with PTD-mediated delivery, luciferase rapidly crosses the plasma membrane, and we quantified the efficacy of PTD delivery in living cells [7]. In the present study, we demonstrated that PTD-Luc was an effective sensor for real-time measurement of intracellular ATP without cell extraction processes, as depicted in Figure 1.

**Figure 2.**
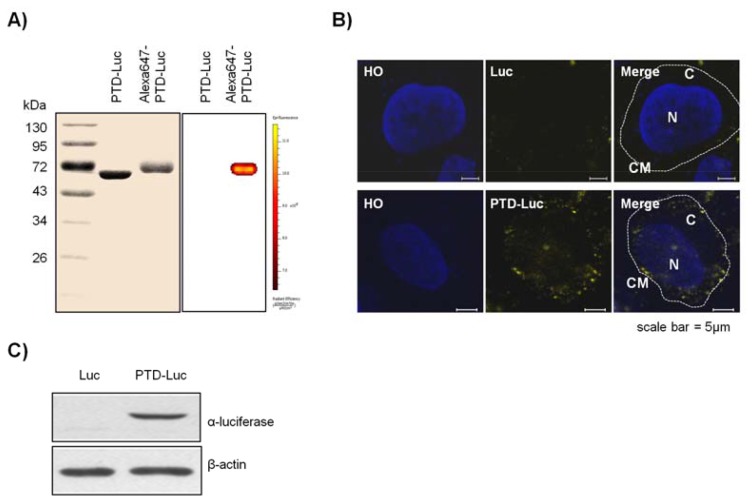
Cellular uptake of PTD-Luc in HeLa cells. (**A**) Characterization of Alexa 647-PTD-Luc, which was separated using SDS-PAGE and then visualized using Coomassie Brilliant Blue R (left) or an IVIS-200 imaging system (right). (**B**) Representative fluorescence image of the cellular uptake of Alexa 647-PTD-Luc in HeLa cells. HeLa cells were incubated for 30 min in glucose-free Ringer solution containing 170 nM Alexa 647-PTD-Luc or Alexa 647-Luc. The nuclei were stained with Hoechst 33342 (HO, inset). Note that the Alexa 647 signal (yellow) was generated within the cells (scale bar, 5 μm). C, cytosol; N, nucleus; CM, cell membrane. (**C**) Representative Western blot of luciferase and β-actin from HeLa cell lysates. HeLa cells were incubated with 40 nM Luc or 40 nM PTD-Luc for 5 min and washed twice with glucose-free Ringer solution. After cell lysis, the presence of intracellular Luc or PTD-Luc was assessed using anti-luciferase antibody.

To confirm the intracellular uptake of PTD-Luc, we first labeled the PTD-Luc protein with Alexa 647 and characterized Alexa 647-labeled PTD-Luc using SDS-PAGE. This resulted in a single band at around 61 kDa for both PTD-Luc and the Alexa 647-labeled PTD-Luc, with a slight band shift for the Alexa 647-labeled PTD-Luc compared with the PTD-Luc protein (Figure 2(A), left panel). In addition, a fluorescence signal was detected only from the Alexa 647-PTD-Luc without free Alexa 647 (Figure 2(A), right panel). To investigate the cellular distribution of Alexa 647-PTD-Luc, HeLa cells were treated with Alexa 647-PTD-Luc for 30 min and subjected to confocal fluorescence imaging. Fluorescence from Alexa 647-PTD-Luc was detected throughout the cell cytosol, whereas Alexa 647-Luc fluorescence was rarely detected in the cytosol, although some was visualized surrounding the cell membrane (Figure 2(B)). This suggests the superior cellular uptake of PTD-Luc compared with Luc, which was confirmed by Western blot analysis of lysates of PTD-Luc-treated cells (Figure 2(C)). These results indicate that the greater cellular uptake of PTD-Luc allows the intracellular luciferase reaction to occur, thereby providing a new method for directly measuring intracellular ATP content, without requiring cell extraction processes.

**Figure 3.**
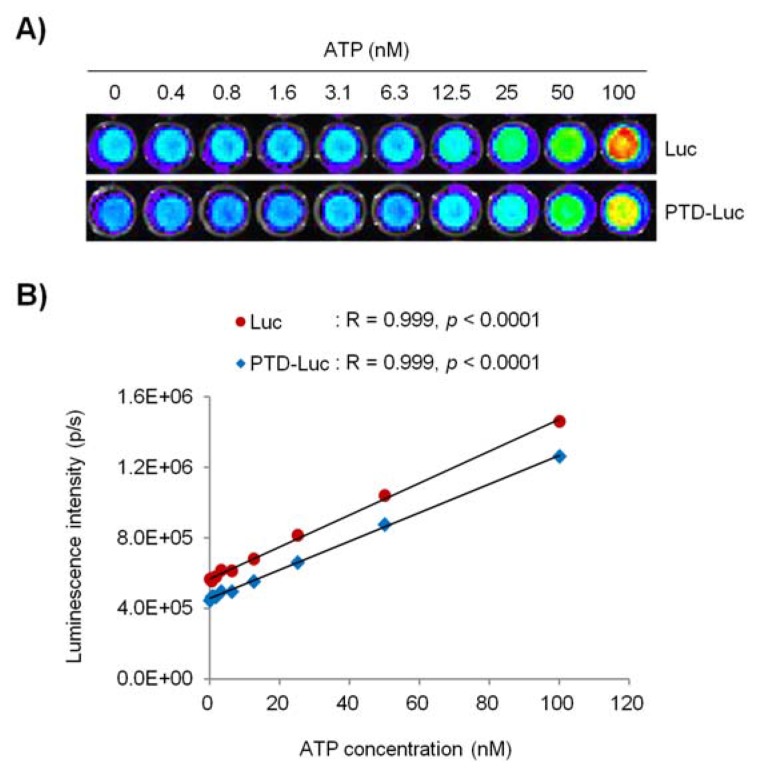
Effect of PTD conjugation on the *in vitro* ATP assay (**A**) Representative luminescence images. PTD-Luc (40 pM) or Luc (10 pM) was incubated with luciferin substrate solution with the indicated concentrations of ATP. (**B**) The correlation between the luminescence intensity (p/s, *y*-axis) and ATP concentration (nM, *x*-axis) is plotted. Symbols represent mean values, and lines represent the linear data fit. Pearson's correlation coefficient (R) is shown above the plot.

Next, we investigated whether PTD conjugation affected the sensitivity and reliability of the luciferase activity in the ATP measurement, by comparing the ATP concentration-dependent linearity of PTD-Luc and Luc using an *in vitro* luciferase assay. PTD conjugation resulted in a significant reduction in the sensitivity of the luciferase reaction to ATP over the entire concentration range tested, as demonstrated by the comparable luminescence signals between 40 pM PTD-Luc and 10 pM Luc. Nevertheless, the sensitivity of PTD-Luc was still sufficient to detect less than 10^–9^ M ATP (Figure 3(A)). Moreover, the luciferase activity of PTD-Luc was proportional to the ATP concentration (R = 0.999, *p* < 0.001; Figure 3(B)), which compares favorably with the reliability of Luc (R = 0.999, *p* < 0.001), suggesting that the PTD conjugation did not affect the reliability of the luciferase reaction with ATP.

**Figure 4.**
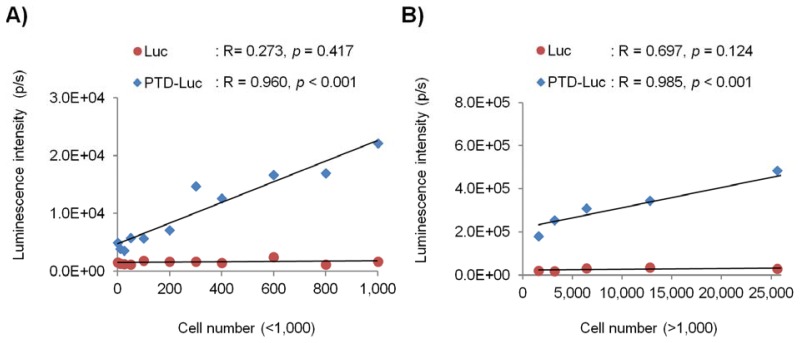
Evaluation of PTD-Luc in the *in vivo* ATP assay. HeLa cells were diluted two-fold serially to give from 0 to 100,000 cells per well and incubated in a 96-well plate in DMEM with 10% FBS. The luminescence signal (p/s) was recorded 5 min after treatment with 40 nM Luc or PTD-Luc. The luminescence signal is plotted against the cell number for 50 to 1,000 cells per well (**A**) and more than 1,000 cells per well (**B**). Symbols represent mean values, and lines represent the linear data fit. Pearson's correlation coefficient (R) is shown above the plot.

To evaluate the *in vivo* sensitivity and reliability of PTD-Luc for intracellular ATP measurement, we investigated the intracellular luminescence signals after PTD-Luc treatment for various cell numbers. HeLa cells were diluted two-fold serially to give from 0 to 100,000 cells per well, and the luminescence was measured 5 min after PTD-Luc or Luc treatment. The luminescence signal of PTD-Luc was 12.6 times that of Luc in 1,000 HeLa cells, and PTD-Luc could measure the intracellular luminescence of as few as 50 cells. Strong correlations were found between the luminescence signal and cell number over a wide range of cell numbers, from high and low (Figure 4, R = 0.985, *p* < 0.001, for >1,000 cells; R = 0.960, *p* < 0.001, for <1,000 cells). These results are consistent with many reports using cell extracts to show that the amount of ATP bioluminescence is directly proportional to the number of cells [2,11,12]. In comparison, the luminescence signal measured with Luc treatment was not significantly correlated with either high or low cell numbers (R = 0.697, *p* = 0.124, for >1,000 cells; R = 0.273, *p* = 0.417, for <1,000 cells), and the luminescence signal was significantly lower than that of PTD-Luc. The low *in vivo* sensitivity and reliability of the control Luc compared with PTD-Luc was obviously explained by the absence of cellular uptake. Thus, although PTD conjugation decreased the *in vitro* sensitivity, it dramatically increased the *in vivo* sensitivity and reliability, highlighting the utility of PTD-Luc for the live cell-based measurement of intracellular ATP.

**Figure 5.**
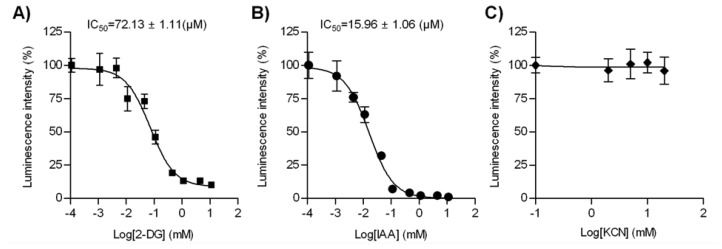
Kinetic analysis of the glycolysis inhibitors 2-DG (**A**) and IAA (**B**), and KCN (**C**). HeLa cells were incubated in glucose-free Ringer solution containing the inhibitor for 1 h, and the luminescence signal (p/s) was recorded 5 min after PTD-Luc treatment (40 nM). The results are expressed as the average percentage luminescence signal relative to the control (no inhibitor). Symbols represent the mean ± SEM, and lines represent the linear data fit. The IC_50_ is given in the plot area. The data were collected from four samples in three independent experiments.

We further validated our live cell-based intracellular ATP assay in a pharmacological application: predicting the cellular toxicity of candidate chemicals in drug screening. To do this, we used three representative metabolic blockers: 2-DG, IAA, and KCN. 2-DG is a non-metabolizable analog of glucose that inhibits hexokinase, the first enzyme catalyzing glycolysis. IAA is a specific irreversible inhibitor of glyceraldehyde-3-phosphate dehydrogenase (G3PDH), the sixth enzyme catalyzing glycolysis [13,14]. Before treatment with the inhibitors, HeLa cells were incubated in glucose-free Ringer solution for 1 h, to ensure the subsequent inhibition of the glycolytic process with 2-DG or IAA [8]. As expected, the inhibition of glucose utilization by 2-DG resulted in a rapid decrease in the luminescence signal of PTD-Luc, with a half maximal inhibitory concentration (IC_50_) of 72.13 ± 1.11 μM (Figure 5(A)). IAA treatment for 1 h also reduced the luminescence intensity (Figure 5(B), IC_50_, 15.96 ± 1.06 μM), showing greater ATP depletion compared with 2-DG. This might have been due to the presumably small amount of G3PDH in HeLa cells, as IAA inhibits it in a stoichiometric fashion [15]. In contrast to the inhibitory actions of 2-DG and IAA, KCN, which inhibits oxidative phosphorylation, had no effect on the intracellular ATP content (Figure 5(C)). These results concur with reports of the absence of an effect of KCN using a FRET-based ATP indicator [3,16]. The different effects of blocking the glycolytic pathway and oxidative phosphorylation may be attributable to the specificity of cancer cells, including HeLa cells, which produce ATP primarily *via* the glycolytic pathway [17,18]. The different ATP response with PTD-Luc in the presence of different metabolic blockers provides a strong tool for conducting detailed kinetic analyses in drug screening.

## 4. Conclusions/Outlook

This study demonstrated that bioluminescent monitoring with PTD-Luc provides an effective system for measuring the intracellular ATP in live mammalian cells or microbial cells [19], with high sensitivity and reliability. The key advantages of the ATP assay with PTD-Luc can be summarized as follows: (1) the inherent sensitivity to intracellular ATP due to luciferase reactions inside cells; (2) the ability to detect ATP without altering the membrane integrity or interfering with cellular metabolism [20]; (3) the capacity for real-time measurement in live cells, enabling detailed kinetic analyses; and (4) the amenability to HTS applications, as there is no need for additional cell extraction processes. Therefore, the live cell-based intracellular ATP assay with PTD-Luc is a useful tool for HTS of the cellular toxicity of large compound libraries in the pharmaceutical industry, with detailed kinetic analysis.
